# Retracted: MiR-451 Promotes Cell Proliferation and Metastasis in Pancreatic Cancer through Targeting CAB39

**DOI:** 10.1155/2021/3702061

**Published:** 2021-03-19

**Authors:** BioMed Research International

*BioMed Research International* has retracted the article titled “MiR-451 Promotes Cell Proliferation and Metastasis in Pancreatic Cancer through Targeting CAB39 [1]” due to an error in sequence targeting.

It was raised to our attention [2] that both the anti-miR oligonucleotides, UUCUCCGAACGUGUCACGUTT and ACGUGACACGUUCGGAGAATT, have no clear target and have been used in many other articles as non-targeting controls. The sequences for si-CAB39, miR-NC, anti-miR-NC, and si-NC were not given in the article. The authors could not be contacted.
